# A novel methodology for fast reservoir simulation of single-phase gas reservoirs using machine learning

**DOI:** 10.1016/j.heliyon.2022.e12067

**Published:** 2022-12-07

**Authors:** Subhrajyoti Bhattacharyya, Aditya Vyas

**Affiliations:** Deysarkar Centre of Excellence in Petroleum Engineering, Indian Institute of Technology Kharagpur, West Bengal, India

**Keywords:** Reservoir simulation, Single phase gas reservoirs, Conventional reservoirs, Rate forecast, Random Forest (RF), Machine-learning

## Abstract

Reservoir simulation is needed for forecasting hydrocarbon production, determining pressure and saturation, well planning, and field development, among other things. The primary objective is to estimate reservoir performance over a period of time and use that data to enhance hydrocarbon recovery under existing operating conditions. In commercial reservoir simulators, a large number of grid blocks are employed to capture the comprehensive information about a reservoir model, such as porosity and permeability, when the reservoir becomes heterogeneous and complicated. This large number of grid blocks is associated with a large number of mass balance equations that need to be solved simultaneously thereby increasing the amount of computational time it takes to solve them. During reservoir simulation, while moving from one-time level to the next requires a large number of iterations if the properties of reservoir fluids are pressure-sensitive. These further increases the computational cost needed during simulation. The primary objective of this paper is to present a novel approach for reservoir simulation that uses Random Forest (RF) which is one of the widely used Machine learning (ML) algorithm to reduce the number of iterations at each time step and speed up the process. This study investigated the benefits of employing the novel approach created using RF with an application to a conventional single-phase gas reservoir. The study's novelty is in developing a new ML-based reservoir simulator that will make reservoir simulation much faster and computationally more efficient. The standard physics-based system of equations has been included while the traditional reservoir simulation algorithm is modified.

## 1 Introduction

### 1.1 Reservoir simulation

Reservoir simulation is one of the most effective tools for integrating data and expertise from various disciplines, including geology, petrophysics, geophysics, reservoir, and production engineering, to model fluid flow in a reservoir. To improve geological understandings and develop predictive capabilities, the reservoir simulation model is history matched using pressure and production measurements from the asset. Because no two hydrocarbon reservoirs are alike and each has its own set of geological properties and drive processes, reservoir simulation and modeling must be customized to each situation to accurately predict the past and forecast the future of a field, (Mohaghegh, 2014).

Reservoir simulation is a multi-step process that starts with building an adequate geological model and ends with estimating field performance, such as gas production rates. The problem is to simulate reservoir dynamics, i.e., time-dependent estimate variables (pressure, well's production rates, etc.) once all the geological factors (starting and boundary conditions) and control parameters are determined. A standard approach is based on the numerical evaluation of a system of hydrodynamic equations, (Illarionov et al., 2022).

The simulation of a reservoir is usually done in several stages including: (i) Define the study's goals; (ii) Gather and validate all reservoir data (iii) Develop a reservoir model (iv) History Matching (v) Perform out prediction simulations. Since this process consumes plenty of time, many researchers have attempted to develop improved approaches to make the standard reservoir simulation algorithm simpler and faster due to the complexities involved, (Ertekin et al., 2001).

### 1.2 Literature survey

A study proposed an ML-based approach to 3D modeling in a development unit. Using accessible data on reservoir parameters and high-performance computing systems, a training set and a meta-model of the development unit was built. The model performed large number of simulations, which was one of this study's significant drawbacks. The model's construction necessitated the use of high-performance computing systems, which increased the cost of the procedure, (Simonov et al., 2018). Another study applied Smart Proxy for a naturally cracked carbonate numerical simulation model with intricate development stages. It was used for automatic history matching in the SACROC Unit's specified study region. The drawback of the study is that the reduction of simulation time is less. Also, this method is limited to a few simulation cases due to oversimplifying the physics involved, (He et al., 2016). In another study, the authors proposed a field-scale performance assessment of shale-gas reservoirs by employing the Fast Marching Method (FMM) to solve for Diffusive Time of Flight (DTOF). The method is very robust and much faster than the conventional numerical simulation. The principal drawback of this study is that the standard physics-based algorithm has been significantly modified, thereby, restricting its applicability to only unconventional reservoirs, (Zhang et al., 2016). Another study presented the integration of FMM with local grid refinements (LGRs) and an embedded discrete fracture model (EDFM) to simulate hydraulic fractures with natural fractures. The method is much faster than conventional reservoir simulation. The study's main drawback is the modification of the standard physics-based algorithm and restriction of its applicability to only unconventional reservoirs, (Xue et al., 2019).

In another study, the Grid-Based Surrogate Reservoir Model (GSRM) was used to reproduce the pressure and saturation distribution across the reservoir at the grid block level and at each time step with reasonable precision. This study necessitates the employment of substantial computational resources, which increases its cost, (Mohaghegh et al., 2012). Another study presented the Bakken Shale's formation and completion parameter ranges and simulated production responses were used to train an Artificial Neural Network (ANN) that could transfer learning to forecast production responses using input parameters from the Eagle Ford Shale. This study employs a long list of complicated steps that take an excessively long time to execute, (Cornelio et al., 2021). In another study, the analysis combined the FMM- succession of steady-state (SSS) method to undertake reservoir simulation and predict gas production in shale gas reservoirs. Because a smaller number of linear equations must be solved at each time step, this method was found to be unconditionally stable and far more efficient than the conventional implicit finite difference method, (Teng et al., 2018). In this study, a fast reservoir simulator was used for the history matching process to obtain insights with consistent flow behavior using production history data in a shorter time. The study involved many complex mathematical steps that required the employment of substantial computational resources, and a significant reduction in computing time is not observed, (Monfaredi et al., 2020).

In a similar study, data preparation and quality assessment, spatiotemporal database formulation, reservoir model design, and Artificial Intelligence via ANN and Data-Driven Analytics were presented as a novel approach to history matching. The main drawback of this study is that it doesn't involve the traditional physics-based approach in predicting results, (Mohmad et al., 2020). In another study, the authors demonstrated the integrated application of the streamline-based three-phase history matching by including water-cut, gas-oil ratio, and bottom hole pressure data. The method is faster than conventional reservoir simulation. The primary drawback of this study is that it is limited to a few models and doesn't consider the physics involved, (Kam et al., 2017). Another study presented a hybrid approach in which the authors employed the reservoir simulation input and output to build an ML model for unconventional field development. The method gives reliable predictions and is much faster than commercial numerical simulation, (Park et al., 2020).

In another study, a data-driven based model was employed to predict the decline curve parameters of Stretched Exponential Decline Model (SEDM) and used it to predict EUR of oil and rate decline of many test wells and as an effective and fast alternative to reservoir simulation in Eagle Ford Shale Oil wells, (Bhattacharyya and Vyas, 2022a). Another study proposed a ML-based model to predict the decline curve parameters of SEDM and used it to predict EUR and rate decline of many test wells and as an effective and fast alternative to reservoir simulation in Eagle Ford Shale Oil wells, (Bhattacharyya and Vyas, 2022b).

### 1.3 Research problem

Solving mass balance equations in their discretized form was a significant challenge in many studies mentioned above. Commercial reservoir simulators generally run a large number of simulations and execute a substantial number of iterations within each simulation. As a result, the simulation process can take a long time to complete. This raises the computational expenses of the procedure and mandates increased computational requirements. In the literature review presented above, employment of substantial computational resources, simulation execution time and in some cases application of algorithms not considering physics of flow through porous media were primary drawbacks of the proposed methodologies.

### 1.4 Objective

In this study, a Machine Learning model-based single-phase compressible (gas) reservoir simulator has been developed to reduce the number of simulations during the iterative process during a time step, thereby saving simulation time. This makes the process much faster and computationally less expensive without compromising with the accuracy of traditional approach involving mass balance equations.

## 2 Reservoir model description

The model consists of a single-phase compressible (implicit) gas reservoir simulator. It consists of a Cartesian grid, and the reservoir is divided into 40 x 40 x 40 grid blocks and the size of each grid block is 30ft x 30ft x 30ft. The rock permeability and porosity will vary in grid blocks and are randomly assigned to each grid block. The fluid viscosity shall vary with pressure. The initial reservoir pressure is 6200 psi. Bottom-Hole Pressure is fixed at 5076 psi. The reservoir consists of a horizontal well at the center with twenty perforations.

## 3 Methodology

A short description of **Random Forest (RF)** is given below.

### 3.1 Random Forest (RF) (Breiman, 2001)

A Random Forest (RF) consists of multiple uncorrelated trees; each of these trees is modeled from a bootstrap subsample of training data and a subsample of predictors. A regression tree is constructed by the repeated partition of variable data space such that the Residual Sum of Squares (RSS) at each node reduces. A training data with replacement is employed from generating a bootstrap subsample of training data. The final prediction in regression trees is given by an averaged response, (Breiman, 2001).

The RSS is calculated by using Eqs. (1) & (2) as shown below

(1) $$\mathrm{RSS}=\sum_{c=1}^{n}\sum_{i=1}^{n_{c}}{\left(y_{i}-m_{c}\right)}^{2}$$

(2) $$m_{c}=\frac{1}{n_{c}}\sum_{i=1}^{n_{c}}y_{i}$$

*c* = no. of nodes
$n_{c}=$no. of data points in a node
$y_{i}=$observed or actual response value

In order to achieve this, each node is split to ensure that the RSS is reduced to the least, and is accomplished by comparing multiple split possibilities using different variables and split points within those variables. When a node is split, two nodes are generated and subsequent splitting is carried out until the number of data points in each node attains the predetermined limit, (Breiman, 2001).

### 3.2 PDE and discretization form for simulation, (Ertekin et al., 2001)

Eq. (3) shows the general PDE for single phase

(3) $$\nabla (\frac{pk}{\mu }(\nabla p+g\nabla z))=\frac{\delta \varphi \rho }{\delta t}+q$$

where,

∇ = lambda operator
p = fluid pressure, psi
k = rock permeability, millidarcy
*μ* = viscosity of oil, centipoise
g = acceleration due to gravity, cm/s^2^
*φ* =  porosity, %
*δ* = delta operator
*ρ* = density of oil, gm/cm^3^
q = oil flow rate, cm^3^/s

Eq. (4) shows the discretized 3-dimensional single-phase flow

(4) $$T_{i-\frac{1}{2}}P_{i-1}+T_{i+\frac{1}{2}}P_{i+1}+T_{j-\frac{1}{2}}P_{j-1}+T_{j+\frac{1}{2}}P_{j+1}+T_{k-\frac{1}{2}}P_{k-1}+T_{k+\frac{1}{2}}P_{k-1}-(T_{i-\frac{1}{2}}+T_{i+\frac{1}{2}}+T_{j-\frac{1}{2}}\;\;+T_{j+\frac{1}{2}}+T_{k-\frac{1}{2}}+T_{k+\frac{1}{2}})P_{ijk}=C_{i}V_{i}\frac{\delta p}{\delta t}+q$$

where,

In the above equation, Eqs. (5), (6) & (7) show the LHS's Transmissibility terms

(5) $$T_{i\pm \frac{1}{2}}=\frac{\frac{0.001127⁎K_{X}⁎D_{Y}⁎D_{Z}}{D_{X}}⁎1}{B_{o}\mu_{o}}$$

(6) $$T_{j\pm \frac{1}{2}}=\frac{\frac{0.001127⁎K_{Y}⁎D_{X}⁎D_{Z}}{D_{Y}}⁎1}{B_{o}\mu_{o}}$$

(7) $$T_{k\pm \frac{1}{2}}=\frac{\frac{0.001127⁎K_{Z}⁎D_{X}⁎D_{Y}}{D_{Z}}⁎1}{B_{o}\mu_{o}}$$

$T_{i\pm \frac{1}{2}}=$Transmissibility at the interfaces of the grid blocks in +/- x-directions
$T_{j\pm \frac{1}{2}}=$Transmissibility at the interfaces of the grid blocks in +/- y-directions
$T_{k\pm \frac{1}{2}}=$Transmissibility at the interfaces of the grid blocks in +/- z-directions
$P_{i\pm 1}=$Pressure in the $i+1^{th}/i-1^{th}$ grid blocks in the x-directions, psi
$P_{j\pm 1}=$Pressure in the $j+1^{th}/j-1^{th}$ grid blocks in the y-directions, psi
$P_{z\pm 1}=$Pressure in the $z+1^{th}/z-1^{th}$ grid blocks in the z-directions.

The subscripts refer to the grid's interface connectivity between the plus and minus orientations. The conversion factor, 0.001127 is to use oil field units in these equations. On the RHS, Eqs. (8) & (9) show the $C_{i}V_{i}$ term i.e. the accumulation term & the discretize form of the equation, respectively.

(8) $$C_{i}=\frac{\varphi }{B}$$

It can be re-written to discretize as

(9) $$C_{i}=\frac{\varphi^{o}c_{R}}{B_{i}^{n+1}}+\frac{\varphi^{n}c_{f}}{B^{o}}$$

Applying finite difference method on LHS, the PDE becomes Eq. (4)

(10) $$T_{i-\frac{1}{2}}P_{i-1}+T_{i+\frac{1}{2}}P_{i+1}+T_{j-\frac{1}{2}}P_{j-1}+T_{j+\frac{1}{2}}P_{j+1}+T_{k-\frac{1}{2}}P_{k-1}+T_{k+\frac{1}{2}}P_{k-1}-(T_{i-\frac{1}{2}}+T_{i+\frac{1}{2}}+T_{j-\frac{1}{2}}\;\;+T_{j+\frac{1}{2}}+T_{k-\frac{1}{2}}+T_{k+\frac{1}{2}})P_{ijk}=C_{i}V_{i}\frac{\delta p}{\delta t}+\text{q}$$

Eq. (11) shows the implicit discretization of the accumulation term

(11) $$\frac{\left(\frac{\varphi^{o}c_{R}}{B_{i}^{n+1}}+\frac{\varphi^{n}c_{f}}{B^{o}}\right)V}{\Delta t}(P_{i}^{n+1}-P_{i}^{n})$$

$B^{o}=$Oil Formation Volume Factor, rb/stb
$\varphi^{o}=$Oil viscosity, %
$c_{R}=$Rock compressibility, 1/psi
$c_{f}=$Fluid compressibility, 1/psi
$P_{i}^{n+1}=$Pressure in $i^{th}$ grid block in $n+1^{th}$ time level, psi
$P_{i}^{n}=$Pressure in $i^{th}$ grid block in $n^{th}$ time level, psi
$B_{i}^{n+1}=$Oil Formation Volume Factor in $i^{th}$ grid block in $n+1^{th}$ time level, rb/stb

Eq. (12) shows the term q in Eq. (4)

(12) $$q=\frac{WI}{B\mu }(P-P_{wf})$$

where,

WI = well productivity index

Eq. (13) shows the well productivity index

(13) $$\text{WI}=\frac{2\pi ⁎D⁎Z\sqrt{}KxKy}{\ln(\frac{r_{o}}{r_{w}})}$$

where,

$P_{wf}=$Bottom Hole Flowing Pressure, psi
$K_{x}=$Permeability in x-direction, millidarcy
$K_{y}=$Permeability in y-direction, millidarcy
*D* = Spacing of each grid block, feet
*Z* = Number of grid blocks
$D_{x}=$Spacing of each grid block in x-direction, feet
$D_{y}=$Spacing of each grid block in y-direction, feet
$D_{z}=$Spacing of each grid block in z-direction, feet
$r_{w}=$well radius, feet

In the case of the isotropic and square grid, Eq. (14) gives the expression for $r_{o}$

(14) $$r_{o}=\text{0.208}⁎D_{X}=\text{0.208}⁎D_{Y}=\text{0.208}⁎D_{Z}$$

**Matrix construction**

The system of equations in finite difference form is acquired. Eq. (15) shows this system of equations in matrix form

(15) $$TX^{n+1}-D\left(X^{n+1}-X^{n}\right)-Q=0$$

where,

D = Accumulation matrix
T = Transmissibility matrix
Q = Sink/Source (flow matrix)
n+1 = superscript for next time level
n = superscript for current time level

**Accumulation matrix [D]**

The **[D]** matrix is organized in block diagonal form with size of (3*NGRID, 3*NGRID) where, NGRID = No. of Grid Cells. It includes accumulation in each of the grid blocks during a time step (from n^th^ time level to n+1^th^ time level).

**Source/Sink term matrix [Q]**

**[Q]** matrix is matrix with size (3*NGRID, 1). It contains flow rate from each of the grid blocks. In the reservoir model used for this study, only one grid block has non zero value of flow rate and therefore this matrix has only one non zero element. In the case of rate control, the injection/production rate can be put in this **[Q]** matrix. If the pressure is controlled, the Q term has to between written in terms of the well flow equation, as shown in the last section. The term that multiplies $P^{n+1}$ must be subtracted from **[T]** matrix.

**Transmissibility matrix [T]**

**[T]** matrix for the single phase 3D reservoir model is of block hepta-diagonal form with size of (3*NGRID, 3*GRID). This matrix contains transmissibility at each interface in the model.

Fully implicit Method has been used to solve the system of equations. All parameters are evaluated at n+1 time level. Eq. (16) shows the implicit discretization of the matrix system of equations

(16) $$T^{n+1}X^{n+1}-D^{n+1}\left(X^{n+1}-X^{n}\right)-Q^{n+1}=0$$

At this point, all parameters are unknown. We have to evaluate them by Newtonian iteration by means of **Jacobian matrix [J]** and **residual matrix [R]**. Eq. (17) shows the residual matrix

(17) $$\left[R\right]=T^{n+1}X^{n+1}-D^{n+1}\left(X^{n+1}-X^{n}\right)-G^{n+1}-Q^{n+1}$$

And **[J]** is sensitivity of **[R]** respect to change in each unknown

The method provides good result, especially, when the time step size is large.

### 3.3 Reducing the number of iterations

It is observed that when a reservoir simulator performs simulations, it performs a large number of iterations while solving equations for the next time level. These iterations generally consume a large fraction of the total simulation time. In this study, an ML algorithm was used to build a model based on the simulated data gathered by the end of the first 30 days. This particular ML model was used to predict the properties of a gas, such as gas formation volume factor, gas viscosity and gas density before the beginning of the next time step. Since the ML model can closely predict the gas properties of the next time step, which are very close to the actual values, it takes a lesser time of the reservoir simulator to solve for the pressure for the next time level. This is the reason for the reduction in the overall simulation time in the designed reservoir simulator, as shown in Table 2. This can save a considerable amount of simulation time and make the simulation process much faster and computationally less expensive.

Steps performed to reduce the number of iterations:

1. Reservoir Simulation is conducted for the first 30 days without the application of ML. Simulation data at the end of the 30 days is gathered to build ML models.

2. The pressure change w.r.t. time in each grid block is used to build one ML model for each grid block (64,000 ML models for 64,000 grid blocks). The input variables for an ML model for a given grid block include the grid block's pressure, the pressures of the neighboring grid blocks and the transmissibilies across the interfaces with the neighboring grid blocks. The output variable is the pressure of the given grid block at next time level (at the end of current time step).

3. Predict the gas properties like gas formation volume factor, gas viscosity and gas density based on the predicted pressure value by ML model. Repeat this process for all grid blocks in the reservoir model.

4. To solve for the pressures for next time level, instead of using starting values of gas properties based on previous time level, use the gas properties calculated using predicted pressures by ML. Proceed forward and solve the fully implicit equations to get the pressure values. Update the pressure values for each grid block.

5. Update the ML model with recent time step simulation data.

6. Repeat process for subsequent time steps by following steps 3 to 5 as given above.

Table 1 shows the machine learning algorithm employed, input parameters, and output parameters from the designed ML model. The model is used to predict the gas properties in each grid block to be utilized for solving pressures of the next time level.

**Table 1:** Machine learning algorithm employed, input parameters, and output parameters from the designed ML model.

Machine Learning Algorithm	Input Parameters to the ML model	Output Parameters from the ML model
Random Forest	Pressure in the grid blocks (at the beginning of a given time step) and Transmissibilities at the interfaces between the central grid block and the 6 neighboring grid blocks (at the beginning of a given time step)	Pressures at the end of a given time step to be utilized to calculate gas properties like gas formation volume factor, gas viscosity and gas density

In this study, since the reservoir model is in 3D, so the grid block system consists of 7 Grid blocks i.e. one central grid block surrounded by 6 neighboring grid blocks.

The Hyper parameters used for the RF based model used for this study are as given below:

1. The maximum no. of decision trees used for this model is 100.
2. The criterion used for this model is RMSE.
3. The maximum depth in a decision tree is allowed until purity is reached.
4. The maximum no. of splits set at each node is 6.

## 4 Results and discussion

Table 2 summarizes the total simulation execution time taken before and after the application of the ML-based (**RF**) Reservoir Simulator.

$$\text{SETRF}=\frac{\text{Total Simulation Execution time taken before the application of Machine learning based model}}{\text{Total Simulation Execution time taken after the application of Machine learning based model}}=\frac{700\;\text{s}}{250\;\text{s}}=\text{3}$$

From the above table, it is clear that through the application of this novel ML-based reservoir simulator, the simulation execution time can be reduced by three times, i.e., the reservoir simulator can be made three times faster. In other words, the reservoir simulation process can become three times computationally more efficient by applying this novel ML-based reservoir simulator.

**Table 2:** Summary of the reduction of time performed by applying the novel machine learning-based reservoir simulator.

Sl No.	Simulator Type	Total Simulation Execution time taken (s)	Total Simulation Execution time saved (s)
1.	Reservoir Simulation without ML	700	450
2.	Reservoir Simulation with ML	250	

**Figure 1:**
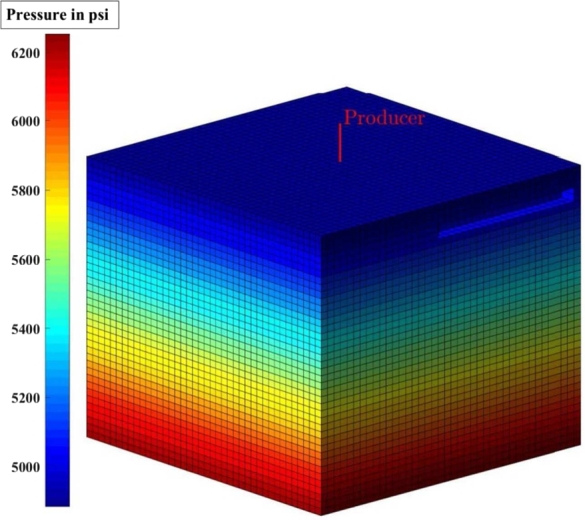
Initial pressure distribution of the reservoir model used for this study.

**Figure 2:**
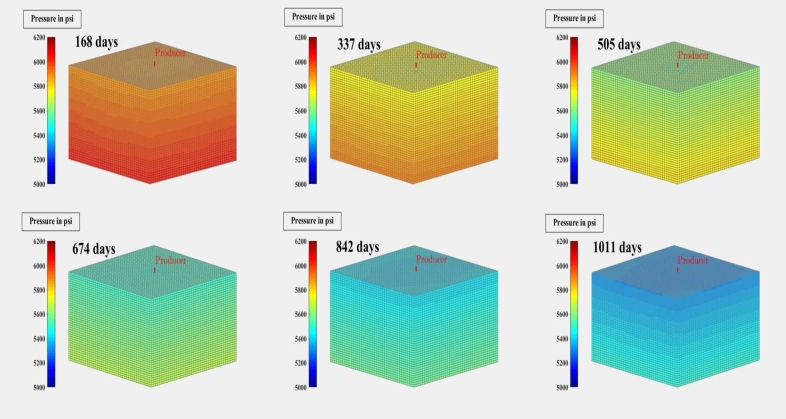
Pressure Distribution in different grid blocks at different time step.

**Figure 3:**
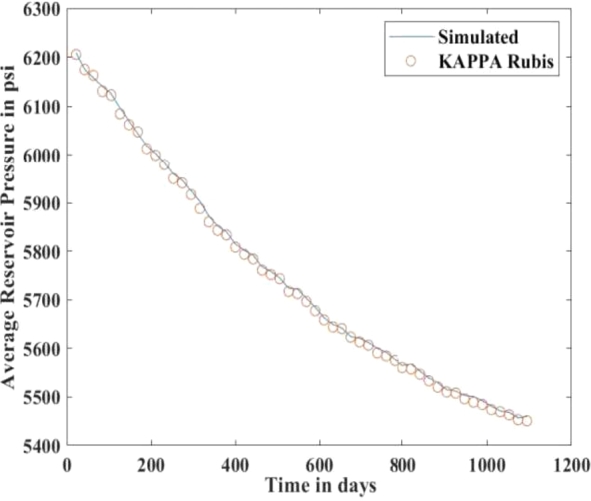
Average Reservoir Pressure.

**Figure 4:**
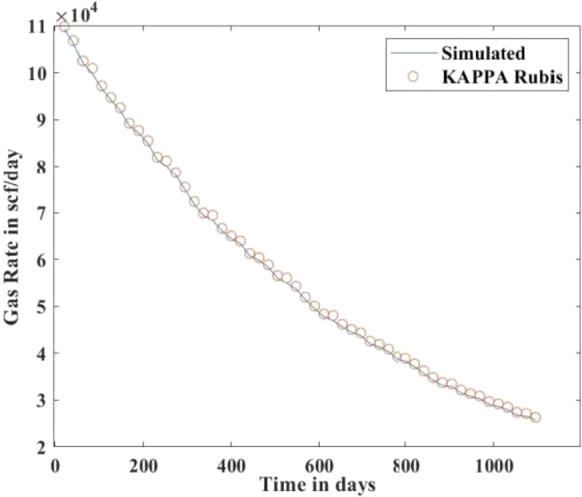
Gas Production Rate.

**Figure 5:**
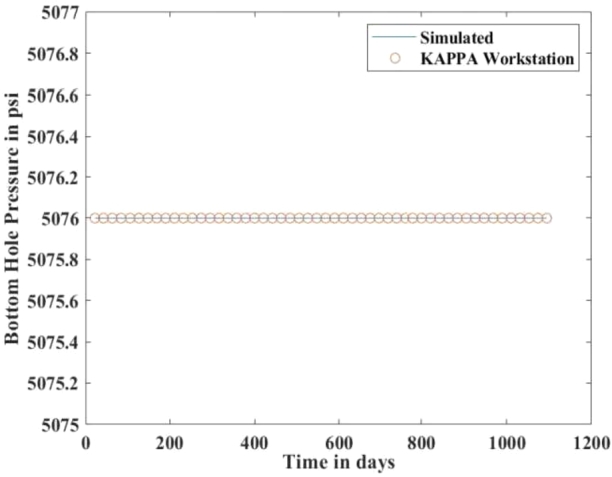
Bottom Hole Pressure.

**Figure 6:**
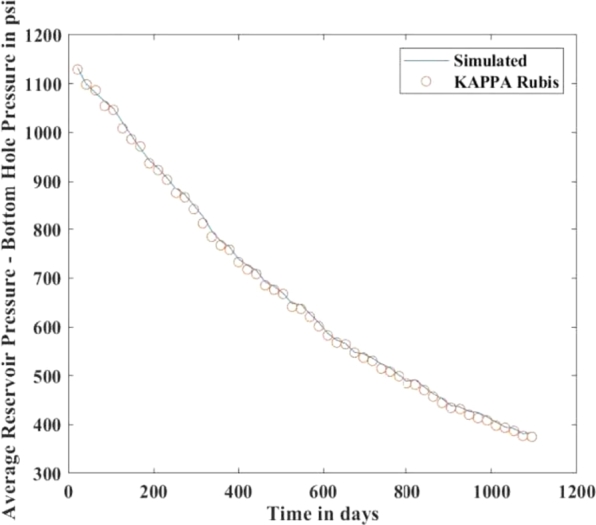
Average reservoir pressure-Bottom Hole Pressure.

**Figure 7:**
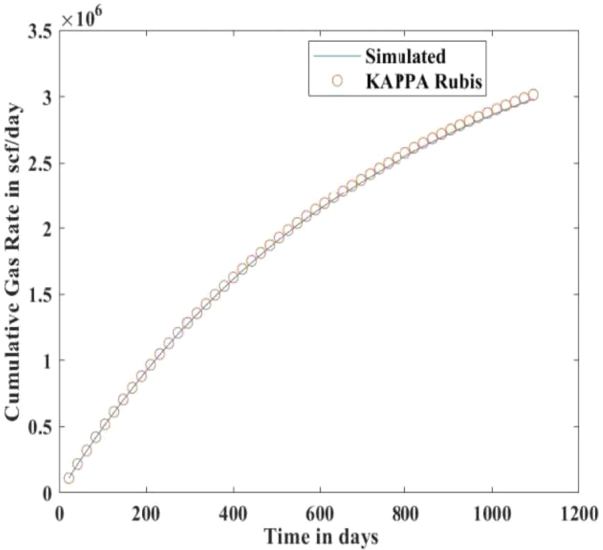
Cumulative Gas Production Rate.

Figure 1, Figure 2, Figure 3, Figure 4, Figure 5, Figure 6, Figure 7 show initial pressure distribution, average reservoir pressure, gas production rate, bottom-hole pressure, average reservoir pressure-bottom-hole pressure, cumulative gas production rate, and pressure distribution in different grid blocks at different time steps. The ML-based reservoir simulator results are compared with those of the **KAPPA Workstation-Rubis** results to determine the accuracy of the ML-based reservoir simulator. In this study, **KAPPA Workstation-Rubis** performs numerical simulation.

By observing the Figure 2, Figure 3, Figure 4, Figure 5, Figure 6, Figure 7, the following conclusions can be made:

Fig. 2 shows the pressure in each grid block at the end of a particular time step, i.e., after 168 days, 337 days, and so on. It helps us to interpret how the pressure changes take place inside the reservoir with the production in time. In this case, the grid blocks at the bottom of the reservoir are more pressurized than the grid blocks at the top of the reservoir due to gravity effects. Fig. 3 shows a declining trend of average reservoir pressure with time. This is expected since, with production in time, the average reservoir pressure declines. This trend is also validated by comparison with KAPPA Workstation-Rubis reservoir simulator. Fig. 4 shows a declining trend in gas production rate with time. This is also expected since, with production in time, the average reservoir pressure declines thereby reducing pressure drawdown. Fig. 5 shows a straight line curve for Bottom Hole Pressure. This is because the Bottom Hole Pressure is fixed as a constraint for this study during entire simulation time. This trend is also validated by comparison with KAPPA Workstation-Rubis. Fig. 6 shows the pressure drawdown which is equal to the difference between average reservoir pressure and well bottom hole pressure. The declining trend with time is evident from Figure 3, Figure 5 because with time as the reservoir depletes, the pressure inside the reservoir declines. This trend is also validated by comparison with KAPPA Workstation-Rubis. Fig. 7 shows the increasing trend of Cumulative Gas Production Rate, which is validated by comparison with KAPPA Workstation-Rubis. From Figure 3, Figure 4, Figure 5, Figure 6, Figure 7, it can be observed that the results of the ML-based reservoir simulator are in close agreement with the **KAPPA Workstation Rubis** reservoir simulator, which indicates that this ML-based reservoir simulator developed for this study can predict the production behavior accurately with minor errors.

## 5 Limitations

In the present study, main objective is to prove the developed methodology. Therefore, its applicability was tested on a synthetic field scale model with single-phase characteristics. In the future, this study can be extended to test the applicability of this methodology for a real field case model such as a standard SPE Model with complex geological heterogeneities. Also, the algorithm developed in this study can be extended to be employed on a multiphase reservoir.

## 6 Conclusions

ML modeling has been successfully employed to develop a novel reservoir simulator that is able to reduce the computational time for reservoir simulation by at least three times. The novel reservoir simulation methodology developed can be particularly useful in practical situations when a very fast prediction of reservoir properties is required within a limited time period and with limited computational resources. The advantage of the developed reservoir simulation methodology becomes more prominent if number of grid blocks in the reservoir model increases.

In the future, this study can be further extended, and the application of this methodology can be verified in 2-phase or 3-phase, highly complicated actual field models like SPE standard models with the employment of other ML algorithms.

## Declarations

### Author contribution statement

Subhrajyoti Bhattacharyya, Aditya Vyas: Conceived and designed the experiments; Performed the experiments; Analyzed and interpreted the data; Contributed reagents, materials, analysis tools or data; Wrote the paper.

### Funding statement

This research did not receive any specific grant from funding agencies in the public, commercial, or not-for-profit sectors.

### Data availability statement

Data included in article/supp. material/referenced in article.

### Declaration of interests statement

The authors declare no conflict of interest.

### Additional information

No additional information is available for this paper.
